# Implementation of an automated methicillin-resistant *Staphylococcus aureus* polymerase chain reaction screening intervention to guide discontinuation of vancomycin for pneumonia

**DOI:** 10.1017/ash.2025.10234

**Published:** 2025-12-01

**Authors:** Reem Azem, Elie A. Saade, Courtney Veltri, Nicholas J. Newman, Natalie Kolehmainen, Khoi Dang, Lisa M. Stempak, Brigid M. Wilson, Leila S. Hojat

**Affiliations:** 1 University Hospitals of Cleveland (Cleveland Medical Center), Cleveland, OH, USA; 2 Case Western Reserve University, Cleveland, OH, USA; 3 University Hospitals of Cleveland (Ahuja Medical Center), Cleveland, OH, USA; 4 Cleveland Clinic, Cleveland, OH, USA; 5 VA Northeast Ohio Healthcare System, Cleveland, OH, USA

## Abstract

**Background:**

Methicillin-resistant *Staphylococcus aureus* (MRSA) is a pathogen commonly implicated in pneumonia for which vancomycin is often used to cover empirically. Our study examined the impact of a pharmacy-based automatic MRSA polymerase chain reaction (PCR) nares intervention.

**Methods:**

We included patients prescribed vancomycin for pneumonia and defined three cohorts: pre-intervention (Cohort 1), post-test implementation (Cohort 2), and post-test implementation with electronic medical record (EMR) enhancements and active stewardship involvement (Cohort 3). The primary outcome was total duration of vancomycin treatment. We performed a desirability of outcome ranking with response adjusted for duration of antibiotic risk (DOOR/RADAR) analysis to prioritize clinical outcomes including adverse events, escalation of care, and death.

**Results:**

The median duration of vancomycin therapy decreased between Cohorts 1 and 2 (55 versus 47 hours, *p* = 0.15), with a greater decrease detected among the subset of Cohort 2 patients from whom the PCR test was actually collected (42 hours, *p* = 0.035). Duration of therapy was lowest in Cohort 3 (22.3 hours, *p* < 0.001). In the DOOR/RADAR analysis, no meaningful difference in clinical outcomes was detected between Cohorts 1 and 2 (*p* = 0.55). Cohort 3 had substantially better ranked outcomes when compared to Cohorts 1 and 2 (*p* < 0.001).

**Conclusion:**

Vancomycin treatment duration was shorter in patients after a pharmacy-directed, EMR-based MRSA PCR intervention was implemented, with a greater response after implementation of additional EMR enhancements and active stewardship involvement, improving test collection rate and result response. This study will help inform future interventions pairing automated EMR-based interventions with stewardship team efforts.

## Key points

- Electronic medical record-based interventions using methicillin-resistant *Staphylococcus aureus* polymerase chain reaction (MRSA PCR) for vancomycin de-escalation in pneumonia can decrease time to vancomycin discontinuation.
- Active stewardship involvement can enhance the efficacy of an MRSA PCR intervention for vancomycin de-escalation.

## Introduction

Methicillin-resistant *Staphylococcus aureus* (MRSA) is a top global multi-drug-resistant pathogen with the greatest global rise in attributable drug-resistant pathogen deaths, increasing from an average of 103,000 deaths in 1990 to an average of 196,000 deaths in 2021.^1^ Guidelines for hospital-acquired and ventilator-associated pneumonia (HAP/VAP) by the Infectious Disease Society of America and American Thoracic Society recommend MRSA coverage for empiric treatment in most patients.^2^ Guidelines for community acquired pneumonia also include recommendations for empiric vancomycin use in certain patients based on local risk factors or prior MRSA infection.^3^

Efforts to reduce vancomycin usage have been a focus of many antimicrobial stewardship programs given its associated toxicities including nephrotoxicity and hypersensitivity reactions,^4^ contribution to increasing rates of resistance as seen in vancomycin-resistant enterococci (VRE)^5^ and less commonly, vancomycin intermediate and vancomycin-resistant *Staphylococcus aureus* (VRSA^6^), and added health care costs and blood draw frequency associated with laboratory monitoring.^7^ Vancomycin also requires substantial personnel time for pharmacy preparation, pharmacy-to dose protocols, and nursing administration. Preparation and administration of vancomycin generates a large amount of solid waste and associated greenhouse gas emissions.^8^

MRSA screening from the nares or throat has an excellent negative predictive value (NPV) of over 90% for MRSA pneumonia,^9^ helping providers rapidly discontinue anti-MRSA therapy in patients with negative screens. Polymerase chain reaction (PCR)-based methods have the advantage of quicker turnaround time compared to traditional culture on chromogenic agar. Variation in median times to results exists with one study citing a median turnaround time of 1.9 hours for PCR results versus 66.9 hours using culture-based methods.^10^

Recent studies have shown the effectiveness of MRSA PCR on vancomycin de-escalation in pneumonia^11^ in various settings including emergency departments^12^ and critical care units.^13^ Pharmacy-based interventions for anti-MRSA agent de-escalation have shown increased vancomycin discontinuation rates when utilizing MRSA swabs; however, clinical outcomes have not been as well studied.^14^ The aim of our study was to examine the impact of a pharmacy-driven, electronic medical record (EMR)-based intervention on reducing vancomycin usage by facilitating early discontinuation of vancomycin in patients hospitalized with pneumonia. Additionally, we evaluated the clinical impact of early vancomycin discontinuation.

## Methods

### Population and ethics

Our population included adult patients hospitalized in a 12-hospital, integrated healthcare system in Northeast Ohio. The study cohort comprised patients who received vancomycin for the indication of pneumonia. The study was approved by the Institutional Review Board of University Hospitals (STUDY20210677).

### Intervention

**Figure 1.**
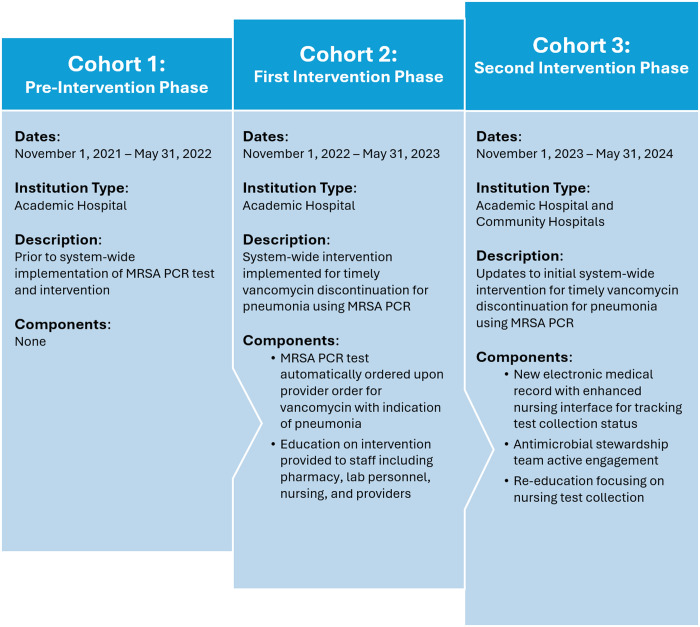
Flowchart of intervention phases with descriptions of key components defining each phase.

We launched a systemwide intervention in August 2022 aimed to assist with timely vancomycin de-escalation in patients with pneumonia (Figure 1). Within our healthcare system, pharmacists manage vancomycin dosing, triggered by an order for pharmacist-directed dosing placed at the time of the initial vancomycin order. At ordering, providers must include clinical indication to ensure appropriate therapeutic targets. In the first phase of our initiative, the MRSA PCR nares test was automatically added when a provider ordered vancomycin and specified the indication of pneumonia. If the vancomycin indication was an alternative diagnosis (eg, sepsis) but the pharmacist had high clinical suspicion for pneumonia, the pharmacist could order the MRSA PCR themselves.

Pharmacists, providers, and nursing staff were educated on the aims of the intervention he test’s high NPV, and importance of prompt collection and response to results. The specific MRSA PCR test utilized was the Cepheid Xpert® MRSA NxG which each hospital validated independently for on-site processing.

Initial implementation review for quality purposes identified that a substantial proportion of tests, despite appropriately ordered, were not collected, secondary to orders falling off the nursing queue. There also appeared to be inconsistent provider response to test results. These observations prompted implementation of the second phase of our initiative. In October 2023, our hospital system adopted a new EMR platform which provided better tools for nursing staff to track orders in their queue. We subsequently reimplemented nursing education to reinforce the critical importance of timely specimen collection. Notably the new EMR system did not allow automatic order entry, forcing a change in the workflow. The revised process involved an alert triggered upon entry of a vancomycin order for the indication of pneumonia, which included a prechecked order for the MRSA PCR screening test. We additionally sought to improve the response time through more active engagement from the antimicrobial stewardship team. Stewardship pharmacists screened for negative results Monday to Friday and performed targeted audit and feedback, notifying teams verbally (in person or via telephone) or by secure message to discuss the recommendation to discontinue vancomycin.

### Study design

We defined three cohorts: Cohort 1 was the preintervention group, which included cases with vancomycin orders placed between November 1, 2021 and May 31, 2022; Cohort 2 was the first intervention phase which included cases between November 1, 2022 and May 31, 2023; and Cohort 3 included cases between November 1, 2023 and May 31, 2024, after the second phase of the intervention was initiated. Only patients who received vancomycin for pneumonia were included, and a patient could only be included once throughout the study. Cohorts 1 and 2 included patients from the main hospital center, a 1,032 licensed bed academic medical center. Cohort 3 included patients from both academic and community medical center sites. Cohort 3 was intended as a prepost analysis for the academic site, and including the community sites also established a cohort for evaluation of the intervention in different settings (ie, Cohort 3 academic site vs Cohort 3 community sites).

Results were also stratified by unit type across cohorts. We prespecified several subgroups, including PCR actually performed and academic versus community sites.

The primary outcome of our study was total duration of vancomycin treatment in hours, from initial ordering time to discontinuation for any reason. To understand the potential clinical impact of the intervention, we performed a desirability of outcome ranking with response adjusted for duration of antibiotic risk (DOOR/RADAR) analysis, a strategy designed to compare the benefit of shorter duration of antibiotic therapy in context of clinical outcomes.^15^ We identified seven ordinal levels of clinical outcomes, from most desirable being no escalation of care, adverse effects, or worsening clinical status to least desirable being death. Escalation of care referred to transfer to a higher acuity unit. Adverse events included acute kidney injury (AKI) per Kidney Disease: Improving Global Outcomes criteria, infusion reaction (or other serious drug reaction), or allergic reaction and were assessed throughout the duration of vancomycin therapy and for 30 days after discontinuation. Clinical status was defined in terms of oxygen requirement, with worsening oxygen requirement referring to changing to a higher modality among room air, low flow, non-invasive high flow, and invasive mechanical ventilation or extracorporeal membrane oxygenation. All clinical outcomes were evaluated within a 30-day period. Cases were ranked first in terms of their clinical outcome level and then by duration of vancomycin treatment in hours, with ranks divided for any ties.

Cases were identified through extraction of vancomycin orders with the indication of pneumonia from the pharmacy administration software database, among which we targeted 125 cases to be randomly sampled for each cohort. Covariates including demographics, comorbidities, hospital type, and unit type were extracted from the electronic data warehouse. Chart review within the EMR was performed to identify treatment duration and clinical outcomes. We also determined the total duration of time between steps in the process of performing PCR screening, including test collection documented by nursing and test resulting in the EMR.

### Statistical analysis

Descriptive analysis was utilized to characterize the demographics, hospital characteristics, unit type, median time to each end point, and clinical outcomes within each cohort. Pairwise Wilcoxon Rank-Sum tests were performed to compare the differences in the median duration of treatment and DOOR distribution between cohorts, after ordering by clinical outcome and duration of therapy, correcting for all ties. Kruskal-Wallis test was also performed for comparison across all 3 cohorts. In addition to a primary analysis comparing all patients in the three cohorts as described above, additional analyses were performed in the prespecified subgroups excluding patients without a PCR test result and comparing the impact of the intervention in the main academic medical center versus the community medical centers, as well as a sensitivity analysis excluding community medical center cases from Cohort 3. All analyses were performed in R version 4.3.2.

## Results

We identified 127, 125, and 117 cases among Cohorts 1, 2, and 3, respectively (Table 1). Most patients were located on general medical-surgical acute care wards at the time of vancomycin initiation, and approximately two-thirds of patients in Cohort 3 were located in the community as opposed to the main academic center.

**Table 1.** Patient and hospital characteristics among each cohort. Values are shown as *n* (percent) unless otherwise specified

		Cohort 1	Cohort 2	Cohort 3	Total
		*n* = 127	*n* = 125	*n* = 117	*n* = 369
Age *		62.0 [51.5, 73.5]	68.0 [54.0, 76.0]	75.0 [66.0, 84.0]	68.0 [59.0, 79.0]
Sex	Female	49 (38.6)	59 (47.2)	67 (57.3)	175 (47.4)
	Male	76 (59.8)	65 (52.0)	50 (42.7)	191 (51.8)
	Other	2 (1.6)	1 (0.8)	0 (0)	3 (0.8)
Race	White	73 (57.5)	73 (58.4)	52 (44.4)	198 (53.7)
	Black	49 (38.6)	45 (36.0)	64 (54.7)	158 (42.8)
	Other	5 (3.9)	7 (5.6)	1 (0.9)	13 (3.5)
Ethnicity	Not Hispanic	123 (96.9)	116 (92.8)	110 (94.0)	349 (94.6)
	Hispanic	2 (1.6)	2 (1.6)	1 (0.9)	5 (1.4)
	Other	2 (1.6)	7 (5.6)	6 (5.1)	15 (4.1)
CCI *		5.0 [3.0, 7.0]	5.0 [4.0, 7.0]	3.0 [2.0, 4.0]	4.0 [3.0, 6.5]
Unit type	Floor	93 (73.2)	98 (78.4)	79 (67.5)	270 (73.2)
	ICU	34 (26.8)	27 (21.6)	38 (32.5)	99 (26.8)
Hospital	Academic	127 (100)	125 (100)	43 (36.7)	295 (80.0)
	Community	0 (0)	0 (0)	74 (63.8)	74 (20.1)

* Indicates values which represent the median with IQR in brackets.

IQR, interquartile range; CCI, Charlson Comorbidity Index; ICU, intensive care unit.

The total duration of therapy for each cohort is summarized in Table 2. The median duration of vancomycin therapy numerically decreased between Cohort 1 and Cohort 2 (55 vs 47 h, *P* = .15), with greater decrease in duration for the subset of Cohort 2 patients for whom the PCR test was actually performed (42 h, *P* = .035); all Cohort 3 PCR tests ordered were sent. The duration of therapy was lower in Cohort 3 overall (22.3 h, *P* < .001 vs both Cohorts 1 and 2) and was lowest for the subset of cases in the community medical center setting in Cohort 3 (12.7 h). In the Cohort 3 community medical center subset, a longer duration of therapy was observed in general medical-surgical floors versus ICU’s (20.4 vs 5.9 h), while limited differences between unit types were apparent in the academic center during any of the three study periods.

**Table 2.** Cohort time endpoints. Cohort 1 refers to preintervention period, Cohort 2 to first postintervention period, Cohort 3 to second postintervention period. Cohort results are in the first 3 columns. The last 3 columns provide subgroup analyses. Times are in median hours with interquartile range in parentheses. Pairwise comparisons of Cohort 1 versus other cohorts total time to vancomycin discontinuation with a p value of<0.05 are in bold. Additional p values for comparisons of Cohort 3 community versus academic sites are included

Population	Time interval	Full Cohort results			Subgroup analyses		
		Cohort 1	Cohort 2	Cohort 3	Cohort 2: PCR sent	Cohort 3: community sites	Cohort 3: academic site
All patients		*n* = 127	*n* = 125	*n* = 117	*n* = 84	*n* = 74	*n* = 43
	Total time from vancomycin order to DC	55.0 (35.3–101.8)	47.0 (26.5–89.5)	**22.3 (6.0–48.8)**	**41.8 (24.1–79.8)**	**12.7 (4.4–47.2)**	**28.9 (16.2–46.4)**
						***P* = 0.006**	
	Order to PCR collection			1.2 (0.6–3.6)	3.5 (1.4, 17.5)	1.2 (0.4–2.8)	1.4 (0.6–3.8)
	PCR collection to result			1.8 (1.4–3.4)	10.3 (6.4, 13.6)	1.6 (1.4–1.8)	3.5 (2.4–4.5)
	Result to DC			18.7 (4.1–40.2)	12.8 (3, 41.6)	9.5 (2.8–38.5)	22.7 (12.2–42.6)
ICU patients		*n* = 66	*n* = 68	*n* = 38	*n* = 48	*n* = 18	n = 20
	Total time from vancomycin order to DC	57 (42.1, 98.4)	44.5 (27.6, 83.1)	**16.2 (6.18–35.5)**	41.5 (24.4, 82.8)	**5.9 (3.7–13.8)**	**29.7 (16.1–45)**
						***P* < 0.001**	
	Order to PCR collection			1.1 (0.6–2.4)	2.8 (1,0, 17.1)	1.0 (.6–1.3)	1.2 (0.55–3.3)
	PCR collection to result			2.6 (1.7–4.1)	10.3 (6.0, 14.0)	1.6 (1.4–1.7)	3.7 (3.1–4.4)
	Result to DC			13.6 (4.3–32.0)	11.3 (2.6, 43.0)	4.5 (2.7–12.4)	24.6 (12.1–43.5)
Non-ICU patients		*n* = 61	*n* = 57	*n* = 79	*n* = 36	*n* = 56	*n* = 23
	Total time from vancomycin order to DC	55 (31.0, 103.5)	52.5 (24.5, 96.0)	**23.3 (6.1–61.3)**	44.0 (24.1, 74.1)	**20.4 (4.7–67.6)**	**25.8 (18.8–47.7)**
						*P* = .2	
	Order to PCR collection			1.4 (.5–4.25)	5.5 (1.5, 18.6)	1.2 (.4–4.5)	1.7 (.7–4.0)
	PCR collection to result			1.7 (1.4–2.6)	10.3 (7.0, 13.1)	1.6 (1.4–1.9)	3.2 (2.4–4.8)
	Result to DC			18.9 (4.2–53.8)	19.5 (4.0, 36.4)	18.2 (3.4–59.1)	21 (12.8–41.4)

DC, discontinuation; ICU, intensive care unit.

**Figure 2.**
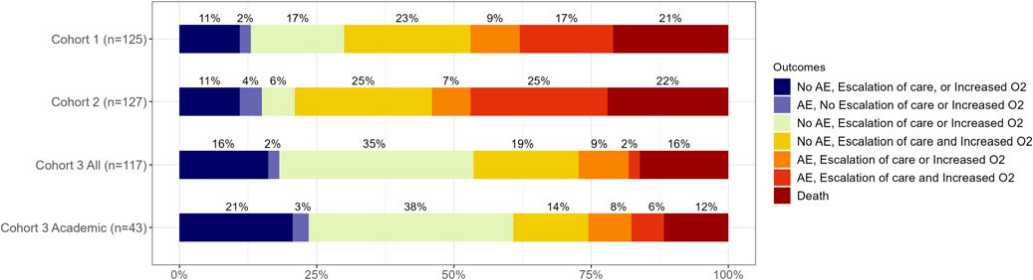
Distribution of the clinical outcome categories among each cohort. Abbreviations: AE, adverse events (acute kidney injury, infusion reaction, or severe antibiotic reaction); O2, oxygen requirement.

**Figure 3.**
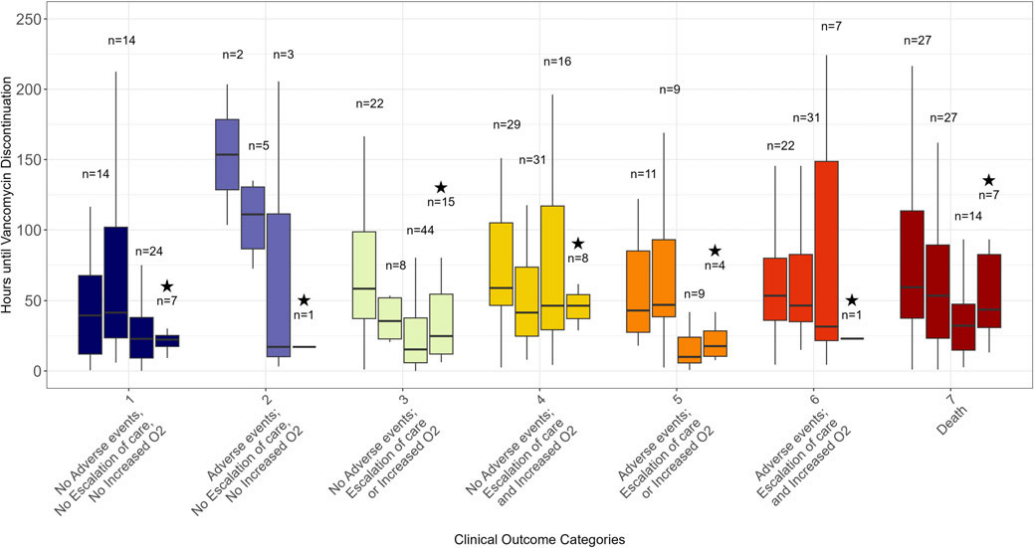
Boxplots for median time to vancomycin discontinuation in hours within each clinical outcome category for both pre- and postintervention groups. Left boxplot within each category corresponds to preintervention group (Cohort 1), second boxplot corresponds to the first postintervention group (Cohort 2), and the third boxplot corresponds to the second intervention group (Cohort 3), and right boxplot designated with a star corresponds to the second intervention group after excluding community cases (Cohort 3 academic). Outliers are not shown. Abbreviations: AE, adverse events (acute kidney injury, infusion reaction, or severe antibiotic reaction); O2, oxygen requirement.

In the clinical outcomes analysis where similar outcomes were ranked according to duration of therapy (DOOR/RADAR), no meaningful difference was detected between Cohorts 1 and 2 (median rank 205.5 vs 232, *P* = .55), even when limiting to Cohort 2 patients with PCR testing performed (median rank 190 vs 197, *P* = .81). However, Cohort 3 had better ranked outcomes when compared to Cohort 1 and 2 patients (median rank 102, *P* < .001), which remained consistent even after excluding community sites (median rank 82 vs 151.1, *P* = .006 [Cohort 1]; 171, *P* = .004 [Cohort 2]). The distribution of outcome levels is shown in Figure 2, which demonstrated overall lower ranked outcomes in Cohort 3, driven largely by a greater proportion of patients with clinical outcome categories of 1 or 3, and fewer with categories 6 or 7. The clinical outcome categories are shown with corresponding median durations of time to vancomycin discontinuation for each cohort in Figure 3. The distribution of treatment duration was much wider in Cohort 3 for several outcome categories with few observations, though this was no longer apparent when limiting Cohort 3 to the academic site. The overall treatment duration was observed to be similar across all outcomes with the exception of categories 2 and 5 which had relatively small numbers of observations.

## Discussion

Our study revealed that overall time to vancomycin discontinuation decreased after implementation of an EMR-based, pharmacy-directed intervention involving MRSA PCR for vancomycin de-escalation, particularly when leveraging active stewardship outreach, enhanced EMR support, and re-education efforts. This outcome is consistent with other published studies describing implementation of pharmacy-based interventions.^14,16,17^ While some studies allowed providers to order the MRSA PCR screen,^17^ other studies^14,18^ restricted these capabilities to pharmacy. Evidence supports the concept that pharmacy oversight and stewardship team involvement enhance the effectiveness of intervention strategies.^19^ By integrating the MRSA PCR order with the pharmacist-directed vancomycin order, such as in our study, pharmacy was able to oversee the intervention, serving as a resource to ensure tests were collected and results acted upon in a timely manner amidst an often-busy work environment.

While the first phase of our intervention succeeded in removing the interval between vancomycin and MRSA screen ordering, this alone did not meaningfully reduce time to discontinuation. The decrease in time to vancomycin discontinuation in our study was most appreciable in Cohort 3, which included patients from the second phase of our intervention after substantial EMR changes and incorporated patients admitted to community medical centers within our healthcare system. Potential factors could have contributed to lower times to vancomycin discontinuation in this cohort, the most likely being the switch to a more user-friendly EMR with a nursing interface less likely to allow orders to be lost. Additional efforts may have added to the intervention’s impact included re-education efforts, intervention familiarity among providers, and increased interaction between our stewardship and medical teams to encourage quicker action. Of note, the time to vancomycin discontinuation was much shorter in the community medical center sites. It is likely that in this setting, lower patient volume and testing load contributed to faster processing and response time.

An essential aspect of our intervention was the education effort. While some studies focus on educating providers only,^11^ our study’s education on the roles and aims of our intervention also included microbiology, pharmacy, and nursing staff. A published review of stewardship efforts to reduce anti-MRSA agents emphasized not only the importance of pharmacy driven efforts, but also provider education in the success of such interventions.^20^ Given that much of this intervention’s effectiveness is dependent on timely collection and processing of the MRSA PCR, nursing and microbiology play integral roles and involving them in education efforts is crucial. After initial observation of collection or processing delays our education efforts were enhanced. An element of individualized clinical decision making is also important to recognize, as seen in cases where vancomycin continued beyond the 48-hour mark despite a negative test. Reviewing indications for ordering MRSA PCR and its utility in clinical practice will help providers incorporate this tool into their everyday practice. The stewardship team made efforts to identify negative tests daily and notified teams of the results, which was particularly useful in cases where results returned after providers had already completed a patient’s daily chart review.

While several studies have addressed the impact of various interventions using MRSA PCR to de-escalate vancomycin use in patients with pneumonia, not all address the question of clinical impact. Thayer et al (2024) looked at length of hospitalization, readmission rates, and mortality, all of which were significantly lower in their postintervention group.^18^ Another study in critically ill patients did not find an appreciable difference in length of stay, 30-day readmission for MRSA infection, reinitiation of anti-MRSA therapy, VRE infection within 30 days, AKI, or in-hospital all-cause mortality.^21^ We examined the clinical impact of early vancomycin discontinuation using DOOR/RADAR, where the most desirable outcome would be no escalation of care, no worsening clinical outcomes, and no adverse effects. While we did not see substantial difference between Cohorts 1 and 2, there were more desirable outcomes in Cohort 3. The reasons for this variation are likely multifactorial. The inclusion of community hospital patients in Cohort 3 likely contributed to lower clinical acuity overall, though our results were similar after excluding community cases. The third cohort additionally coincided with increased stewardship outreach, boosted intervention awareness, and more EMR support.

There are limitations to our study. We utilized a retrospective study design, which relies on the integrity of documentation within the medical record. Due to the change in our health system’s EMR platform, although the process remained the same, differences in functionality of the automated ordering and pharmacy-directed vancomycin dosing processes may have introduced minor differences in timing of initiation and discontinuation of orders. While the third cohort population differed from the second in that it included community hospital patients, it allowed for examination of the intervention using different approaches and different contexts and findings were similar even after excluding these sites. When applying such interventions it is important to consider any given institution’s MRSA PCR screening volume, community MRSA positive colonization rates, and potential selection bias in that patients empirically treated with vancomycin are those at higher risk for MRSA colonization based on risk factors. At our institution, approximately 80 MRSA nares PCR screening tests are sent per month with about 13% positivity, a percentage higher in comparison to the United States (US) population based on a 2012 study that reported approximately 7% of patients in the US are colonized with MRSA.^22^

Future studies should continue to explore the utility of MRSA PCR in other disease processes where it may have good negative predictive value. Stewardship interventions that reduce unnecessary vancomycin use may be associated with reductions in associated personnel time, costs, and waste and may lead to better clinical outcomes.

## Conclusion

Vancomycin treatment duration was shorter in patients after a pharmacy-based automatic MRSA PCR screening intervention was implemented, particularly when enhanced with improved education efforts, increased EMR support, and active stewardship engagement, which in turn may lead to better clinical outcomes in patients. This study will help inform future education efforts for such stewardship interventions, as well as guide future interventions combining rapid testing, EMR tools, and active stewardship response.

## Data Availability

Data from this study is not publicly available but may be available from the corresponding author upon reasonable request.
